# The Lunatic Asylums of India

**Published:** 1900-05-05

**Authors:** 


### The Institutional Workshop.

### THE LUNATIC ASYLUMS OF INDIA.

### MADRAS PRESIDENCY.

The total number of patients who remained under treatment in the three lunatic asylums of the Madras Presidency —Madras, Vizagapatam, and Calicut—at the end of the year 1898 was 577, of which 442 were civil or military lunatics, and 135 belonged to the criminal classes. The proportion of male inmates was largely in excess of the female, the women's side only having 135 occupants, against 442 in the men's quarters. Of these 42 were Europeans. It is an interesting fact that the statistics for the year show a steady increase in the epileptic population of the asylums, although mania is still the most prevalent type of insanity. Intoxicants, according to the statements made, do not seem to have been the cause of the failure of mental power so often as during the previous twelve months, but this is probably owing rather to the more careful sifting of evidence than to any appreciable decrease in ganja-smoking or other forms of excess. The usual large proportion of cultivators is apparent amongst the list of the former occupations of the patients, but there is a decrease in the total of beggars admitted. Although the number of deaths during the year declined, the daily average of sick in the hospital was higher, tubercle of the lungs, which had almost disappeared in 1897, again being responsible for a great deal of illness, and in several cases the disease terminated fatally. The asylums were remarkably free from epidemics, none having invaded either Vizagapatam or Calicut, whilst at Madras there were 32 cases of chicken-pox only, all of which were of a slight character. The matron and three nurses in charge of the sick at Madras rendered good service. The matron and head nurse attended entirely to the women, the other two nurses being employed alternately as day and night nurses in the male hospital. In Vizagapatam and Calicut no separate nursing establishments are maintained, but a certain number of ordinary employés are utilised as hospital attendants. Every effort is made to give the inmates of the asylums both occupation and recreation, agricultural work, weaving, and gardening being the principal employments undertaken by the men, while the European and Eurasian women are formed into a sewing class. In the Madras Asylum the lunatics have a monthly treat with a band, at which light refreshments are provided. There are occasional magic lantern exhibitions, a special entertainment at Christmas, and outdoor and indoor games—football, quoits, bowls, draughts, and cards—are provided. At Vizagapatam and Calicut occasional acrobatic performances are arranged, and sometimes the best-behaved of the lunatics are allowed to go and see a native drama. In the Madras Asylum special clothing has been introduced with much success for the destructive maniacs. It is made of canvas drill and gunny, and is not removable by the wearers. These garments have proved useful in restraining the patients so dressed, and in some cases they have restored a sense of decency, and the desire for clothes. Three of the lunatics succeeded in escaping from Madras, four from Calicut, and one from Vizagapatam during the year. Five of these were recaptured, the rest are still at large.

### LAHORE AND DELHI ASYLUMS.

The number of patients remaining in the Lahore Asylum on December 31st, 1898, was the largest yet recorded since the existence of the institution, viz., 291. It is probable that the increase both here and at the Delhi Asylum is due to the greater readiness on the part of the magistrates to pass orders for the admission of lunatics to asylums, and also to greater willingness on the part of friends to place patients under treatment, rather than to any serious addition to the number of insane persons in the province. The large total of cases admitted in 1898, in which insanity was stated to be due to alcohol drinking, is a somewhat remarkable feature. The number of criminal lunatics admitted to both asylums fell off greatly in 1898, but there has been a steady rise at Lahore in the daily average strength under this head. In fact, the criminal class of lunatic has continued to increase in number until it has now assumed the proportion of one-fourth of the whole number of inmates in the Lahore Asylum. It will be a great boon when the new building at Lahore to accommodate the patients of both asylums is ready for occupation, for the existence of unhealthy overcrowding is demonstrated not only by the somewhat limited amount of superficial feet which it is possible to allow each patient, but also by the fact that there have been twenty-one cases of pneumonia during the year, as against three in the previous year. A large percentage of these cases occurred amongst women, and it is the female portion of the asylum which is the most restricted. The opinion of the Lieutenant-Governor does not, however, agree with the idea that the disease has been the result of want of better accommodation. Having respect to the jail reports, he believes that the pneumonia has been caused simply by the unusual inclemency of the weather. Of the 291 patients in the Lahore Asylum, thirty-six were discharged cured, and thirteen improved. That so many should have benefited is particularly satisfactory, considering that neither here, nor at Delhi, does there appear to be the slightest effort made to provide amusement or recreation of any sort for the inmates. Three of the patients escaped from Lahore during the twelve months, but two were recaptured, the criminal alone succeeding in escaping. There were fifteen readmissions, but of these four were recaptures of escaped lunatics; and three had been absent for two years and over. One woman had been sent to her friends, but after seventeen days had wandered back again of her own accord, finding the society of her relatives uncongenial; and a charas smoker had relapsed again into his evil habits, and been brought back insane, although when he was sent away from the asylum, after four months' treatment, he had quite recovered his mental equilibrium. At Delhi the numbers on the books at the end of 1898 was also higher than any previous record, 133. The percentage of cures decreased, the percentage of deaths increased, being more than any year during the previous decade. According to the reports, no special cause can be assigned for this alarmingly heavy mortality, only three deaths being attributed to pneumonia. The warders are to be congratulated upon their vigilance, as none of the patients succeeded in making their escape. The cost of maintaining the lunatics was greatly reduced, owing to the fall in the price of food-stuffs.

